# Community and individual sense of trust and psychological distress among the urban poor in Accra, Ghana

**DOI:** 10.1371/journal.pone.0202818

**Published:** 2018-09-27

**Authors:** Mawuli K. Kushitor, Maame B. Peterson, Paapa Yaw Asante, Naa Dodua Dodoo, Sandra Boatemaa, Raphael Baffour Awuah, Francis Agyei, Lionel Sakyi, F. Nii-Amoo Dodoo, Ama de-Graft Aikins

**Affiliations:** 1 Regional Institute for Population Studies (RIPS), University of Ghana, Accra, Ghana; 2 Department of Psychology, University of Ghana, Accra, Ghana; 3 Centre for Migration Studies, University of Ghana, Accra, Ghana; London School of Hygiene & Tropical Medicine, UNITED KINGDOM

## Abstract

**Background:**

Mental health disorders present significant health challenges in populations in sub Saharan Africa especially in deprived urban poor contexts. Some studies have suggested that in collectivistic societies such as most African societies people can draw on social capital to attenuate the effect of community stressors on their mental health. Global studies suggest the effect of social capital on mental disorders such as psychological distress is mixed, and emerging studies on the psychosocial characteristics of collectivistic societies suggest that mistrust and suspicion sometimes deprive people of the benefit of social capital. In this study, we argue that trust which is often measured as a component of social capital has a more direct effect on reducing community stressors in such deprived communities.

**Methods:**

Data from the Urban Health and Poverty Survey (EDULINK Wave III) survey were used. The survey was conducted in 2013 in three urban poor communities in Accra: Agbogbloshie, James Town and Ussher Town. Psychological distress was measured with a symptomatic wellbeing scale. Participants’ perceptions of their neighbours’ willingness to trust, protect and assist others was used to measure community sense of trust. Participants’ willingness to ask for and receive help from neighbours was used to measure personal sense of trust. Demographic factors were controlled for. The data were analyzed using descriptive and multivariate regressions.

**Results:**

The mean level of psychological distress among the residents was 25.5 (SD 5.5). Personal sense of trust was 8.2 (SD 2.0), and that of community sense of trust was 7.5 (SD 2.8). While community level trust was not significant, personal sense of trust significantly reduced psychological distress (B = -.2016728, t = -2.59, p < 0.010). The other factors associated with psychological distress in this model were perceived economic standing, education and locality of residence.

**Conclusion:**

This study presents evidence that more trusting individuals are significantly less likely to be psychologically distressed within deprived urban communities in Accra. Positive intra and inter individual level variables such as personal level trust and perceived relative economic standing significantly attenuated the effect of psychological distress in communities with high level neighbourhood disorder in Accra.

## Introduction

Sub-Saharan Africa (SSA) is fast urbanizing, and Ghana is no exception [1,2]. The high influx of migrants from rural to urban areas has resulted in the growth of urban poor and slum communities in the capital, Accra [3]. Within these urban poor and slum communities there is the rising burden of mental health disorders. Mental health disorders such as depression are known to seriously affect the quality of life and increase the risk of premature mortality [4–6]. In addition, mental health problems are strongly associated with chronic conditions and recurrent infectious conditions [7,8]. The double burden of infectious and chronic diseases in SSA is reported to affect urban poor communities disproportionately, and a rising burden of mental health disorders will only worsen the low quality of life of urban poor communities. Community based stressors such as a high crime rate, lack of employment, poor sanitation, mistrust and suspicion have been implicated in the high burden of mental disorders in urban poor communities [9–12]. Some studies have suggested that social cohesion may hold the key to reducing the burden of mental health challenges amongst deprived urban residents.

The relationship between social cohesion and mental health disorders is complex. While several studies have found a positive relationship between constructs of social cohesion such as the sense of trust and belonging, and psychological distress [13–18], other studies have observed a negative relationship [11,13,14]. Social cohesion is a broad concept spanning community sense of belonging, trust in the community, reciprocity (which is a sense of obligation to help others and confidence in return of assistance), equality with community members and community networks. Studies on the urban poor study context usually select ‘sense of trust in the community’, as a dominant variable. Researchers, such as Korayi and Rothermund (2012) demonstrate how trust acts as a resource that is activated in times of need. Others have suggested that the construct of cohesion can infer negative effects on people including, but not limited to, their psychological well-being [19–21]. For instance, we can infer that, while relationships with community members could potentially be beneficial in terms of receiving help, they could at the same time impose a burden on community members who are expected to provide continuous help.

The evidence from the literature highlights implications of factors including poverty, education, age, marital status, employment status, urban/rural residence, household living arrangements, social capital among other factors, on mental health [22–29]. Evidence of community level factors and their impact on mental health remains scarce. A set of extensive reviews of the literature shows that trust is the material from which community safety nets are woven [9,10,30–34]. Our study focused on three urban poor communities in Accra, where community level factors such as crime, insecurity, and poor environmental conditions predispose people to poor individual level psychological outcomes such as anxiety and depression [11]. Using data available from the Urban Health and Poverty Project (EDULINK)–situated in these three communities—we investigate the relationships between community and personal trust and psychological wellbeing, allowing us to test for associations and pathways through which these associations operate.

This study addresses three questions: (1) do community level of trust and individual level sense of trust have an influence on the mental well-being of the urban poor? (2) how do community level sense of trust and individual level sense of trust independently and together influence psychological distress of residents in urban poor localities in Accra? (3) do urban poor residents’ perceptions of relative economic status in society mitigate the relationship between their sense of trust and mental health?

### 1.1 Sense of trust, mental health in the context of urban poverty

Researchers observe that neighbourhood disorder is a function of both mistrust and powerlessness [32,33,35]. Powerlessness results from an inability to control negative community conditions such as a high crime rate and danger. Powerlessness is associated with anxiety, depression and other negative mental health outcomes [11,30–32,36,37]. Mistrust, on the other hand, focuses on the relationship between place and people. Mistrust develops amongst people who have limited social and economic resources and live in contexts where resources are scarce. Places with very high levels of neighbourhood disorder are also associated with high levels of threat. The perceived threat in the community undermines the health [21,38] of community members. Individuals in disadvantaged neighbourhoods are challenged by material deprivation which influences their mental health status. Ross (2011) thus argues that people who live in disadvantaged communities lose their sense of control to neighbourhood disorder which makes them more vulnerable to adverse mental health problems such as psychological distress. There is evidence that community disorder, anxiety and disadvantage are associated with powerlessness and mental health outcomes [26–28].

Positive social cohesion has been associated with positive community outcomes such as care, reciprocity and affection from community members [10]. The effect of mistrust and powerlessness therefore is that people isolate themselves from the broader community and therefore do not receive the benefits of positive social cohesion. Besides isolation from the broader community, mistrust has also been associated with suspicion within the community [39–41]. Communities with high neighbourhood disorder have also been associated with a high sense of suspicion. Suspicion makes people believe that their neighbours will harm them if they had the opportunity. The tendency to be suspicious in neighbourhoods with high levels of perceived social threat makes people protect their vulnerabilities from their neighbours. For instance, people may not disclose their disease status, fearing that in a moment of crises disclosure might be used against them. Psychological distress may be compounded in a situation where people do not talk about important personal issues that concern them. In addition, when people live in a constant state of suspicion it has the potential to affect their mental health in the long term.

Trust enables people to build positive relationships with each other and amongst community members. Trust enables people to share and receive from each other within a given community [42]. Trust may therefore have the potential to mitigate the effect of psychological distress in disadvantaged or urban poor communities. However, very few studies have been conducted to assess the influence sense of trust has on psychological distress in an urban poor context in SSA. Community trust as applied in this study relates to the extent to which people can rely on each in the community. It measures a community level attribute of reciprocity and dependability. Individual trust on the other hand, specifically measures resources available to individuals when they need emotional support from within the community and/or outside of the community.

## Materials and methods

### 2.1 Study setting

The sites for this study were three indigenous communities in central Accra. James Town and Ussher Town are a twin community commonly known as Ga-Mashie, dominated by an indigenous Ga ethnic group. The third site, Agbobloshie, is a heterogeneous migrant slum settlement adjoining Ga Mashie. The prevailing language spoken in the three communities is Ga. However, due to the high influx of internal migrants from other ethnic representations, other languages including Twi, Hausa and Ewe, are spoken in the communities.

The urban poor study area is populated largely by traders and fishermen. This densely populated area is characterized by restricted access to public amenities. For example, the communities have shared public facilities, such as toilets and baths. Characteristic of urban poverty, unemployment and underemployment rates are high in these communities and educational attainment is low [43]. Many community members are engaged in the informal sector sales and services (trading), and manual labour. Other urban poor community dynamics include poor sanitation, a double burden of infectious and chronic diseases and high levels of crime, sex work and drug peddling. These characteristics of urban poverty, as has been shown in several studies in the sub-region have negative implications on security, sense of community and consequently, the health of inhabitants.

### 2.2 Data

This study was carried out with secondary data from the Urban Health and Poverty survey (Edulink Wave III) in Accra. This population-based cross-sectional survey, carried out from September to November 2013, was set in the three urban poor communities -Agbogbloshie, James Town and Ussher Town -as part of the Population Training and Research Capacity for Development (PopTRCD) project that aimed to improve training in the population sciences, as well as study the demography of urban poor communities in Accra, Ghana. Data were collected from women and men aged 15–49 years and 15–59 years respectively, based on a sampling frame designed by the Ghana Statistical Service for the Ghana Demographic and Health Surveys. The sample was drawn from 29 enumeration areas (EAs), each with 40 households systematically chosen to make up a total of 1160 households distributed over the three localities. The number of EAs and, therefore, households in each locality was proportionate to the population size of that locality. There were five EAs from Agbogbloshie, eight from James Town and sixteen from Ussher Town. Six hundred and seventy-five (675) households were selected for the EDULINK survey, yielding a sample size of 782 respondents who were eligible for the individual questionnaire. This sample was used for the analyses of the relationship between respondents’ community sense of trust, personal trust and psychological distress within the context of urban poverty.

The survey instrument addressed a variety of issues, including neighbourhood contexts and psychosocial/mental health. The data were collected using an interviewer-administered questionnaire in English and mostly Ga and Twi, these being languages indigenous to the study area. Forward and backward translation of the questionnaire was done to validate for appropriateness of wording and potential misinterpretation. Interviewers were trained, and the questionnaire was pre-tested prior to the survey.

### 2.3 Ethics statement

Written informed consent was obtained from all respondents prior to conducting the interview. Respondents who could not append their signatures were provided ink pads to thumb print in the relevant sections of the informed consent forms. For participants below the age of eighteen, written consent was signed by parents/guardian. In addition to parental consent, they were required to provide written consent before interviews were conducted. Further, study participants had the option of withdrawing from the study at any point without consequences. Ethical clearance for this study was obtained from the Noguchi Memorial Institute for Medical Research’s Institutional Review Board (NMIMR-IRB) in September, 2013, with the most recent renewal in September, 2017. The protocol number is 105/12-13.

### 2.4 Measures

Evidence on community as well as individual level factors in the extant literature suggests how community sense of trust and individual level background characteristics influence mental health [44]. Incorporating variables from this perspective, the study operationalizes both community and interpersonal level predictors. For the dependent variable, the survey incorporated the Kessler Psychological Distress Scale. This is a symptomatic psychological distress/ wellbeing item scale [45], where respondents were asked about how they had been feeling during the past four weeks. For each question (Table 1), they were asked to give the one answer that came closest to the way they had been feeling, on a 5-point scale ranging from 1 (none of the time) to 5 (all of the time). The scale was scored using the weighted sum of respondents’ answers. On the five-point scale, respondents’ scores ranged from a minimum of 9 to a maximum of 45 for psychological distress.

**Table 1:** Psychological distress item scale.

a. Have you been a very nervous person?
b. Have you felt so unhappy and not yourself that nothing could cheer you up?
c. Have you felt downhearted and let down?
d. Did you feel worn out?
e. Have you felt worthless or hopeless?
f. Did you feel full of life and bounce?
g. Have you felt calm and peaceful?
h. Did you have a lot of energy?
i. Have you been a happy person?

Source: Edulink Wave III, 2013

The conceptualisation of sense of trust is measured from a multilevel perspective; at the community, interpersonal and intrapersonal levels. According to Ross (2011), loss in community sense of trust develops in places where resources are scarce. Alluding to the urban poor context of the study, people may likely develop mistrust in community safety nets in adverse situations, that may result in poor mental well-being. Thus, to explore this relationship to mental well-being, community sense of trust was operationalised as a scale incorporating respondents’ summed answers to three following questions on a Likert scale (1 = strongly disagree; 2 = disagree; 3 = agree 4 = strongly agree): (1) People in this community are willing to help each other; (2) People in this community can be trusted; (3) People in this community watch out for each other. A Cronbach’s alpha of 0.71 indicating a reliable scale was noted. A high score indicates a higher level of trust [13].

Likewise, personal trust was operationalised as a scale incorporating respondents’ summed answers to three following questions on a Likert scale (1 = very likely; 2 = likely; 3 = not very likely 4 = never): (1) How likely are you to ask for help from a neighbour if you needed to talk about something worrying you? (2) How likely do you think you would be able to receive help from a neighbour if you asked to talk about something worrying you? (3) How likely do you think you would be able to receive help from a neighbor outside the community if you asked to talk about something worrying you? Personal trust response was reverse coded. A Cronbach’s alpha of 0.72 was noted. A high score indicates a higher level of personal sense of trust.

Demographic variables controlled for included age, sex, level of educational attainment (primary, JHS, SHS, higher), religiosity operationalised as the number of times a person attended religious services in the past month, locality of residence (James Town, Ussher Town, Agbogbloshie), marital status (in union or not), employment status (employed or not), ethnicity (Ga, Akan, other).

### 2.5 Analytical procedure

The data are analyzed using descriptive statistics (frequency distributions, means and standard deviation), bivariate tests of associations (cross-tabulations, oneway ANOVA), and multivariate regressions. The background characteristics of respondents, including age, sex, marital status, ethnicity, highest level of educational attainment, religious affiliation, locality of residence, type of occupation engaged in, are described using frequency distributions. Bivariate tests of associations between community sense of trust and the other variables, are carried out, using one-way ANOVA. Multilevel regression analysis explores community level and personal sense of trust effects on psychological distress outcomes. Here, multiple linear regression is employed due to the continuous nature of the dependent variable.

## Results

### 3.1 Characteristics of the participants

The demographic and socio-economic characteristics of the participants are presented in Table 2. Females constituted a little more than half of the participants (56%). Majority (45%) of the participants had attained junior high school education. Approximately 6% of the participants had tertiary education. About two thirds (65%) of the participants were employed. With regard to marital status, more than half (55%) of the participants were single. The mean score for psychological distress was 25.4. Overall, the mean score for individual-level sense of trust was 8.2, while that of community-level sense of trust was 7.5 (see Table 2).

**Table 2:** Demographic characteristics of respondents.

Demographic Characteristics	Frequency (%)
**Gender**	
Male	344 (44.0%)
Female	438 (56.0%)
**Education**	
Primary school	137 (18.7%)
Junior high school	332 (45.2%)
Senior high school	223 (30.4%)
Tertiary	42 (5.7%)
**Locality**	
James Town	218 (27.9%)
Ussher Town	457 (58.4%)
Agbogbloshie	107 (13.7%)
**Employment Status**	
Employed	510 (65.2%)
Unemployed	272 (34.8%)
**Marital Status**	
Married	156 (19.9%)
Cohabiting	182 (23.3%)
Single	434 (55.5%)
Psychological Distress	25.35 (±5.5)
Individual-level sense of trust	4.93 (±1.9)
Community-level sense of trust	7.47 (±2.1)
Perceived economic standing	2.64 (±1.9)
Age	30.32 (±10.6)

### 3.2 Relationship between demographic characteristics and psychological trust

Table 3 describes the bivariate relationship between demographic characteristics and psychological distress. Oneway ANOVA was used because the dependent variable was continuous. The bivariate table shows that among the demographic characteristics, sex, education and perceived relative economic standing were significantly associated with psychological distress at the bivariate level. The mean psychological distress score was lower for males than for females. In terms of education, as the level of education attainment increased, psychological distress decreased. A similar negative relationship was observed for perceived relative economic standing. Locality, marital status, employment status, religiosity and ethnicity were not significantly associated with psychological distress.

**Table 3:** Relationship between demographic characteristics and psychological distress.

Variables	Mean	SD	F stats	Prob > F
**Locality**			2.61	0.0746
Usshertown	25.71	5.34		
Jamestown	24.67	5.90		
Agbogbloshie	25.54	5.39		
**Sex**				
Male	24.69	5.62	9.48	0.0021
Female	25.92	5.38		
**Education**			5.83	0.0006
Primary	26.74	5.36		
Middle/JSS	25.39	5.72		
Secondary	24.78	5.72		
Higher	23.26	5.20		
**Marital Status**				
Not in Union	25.55	5.72	0.95	0.3000
In Union	25.16	5.22		
**Religiousity**				
Low	25.6	5.45	0.9	0.4073
Medium	25.08	5.42		
High	25.64	5.68		
**Ethnicity**				
Ga	25.12	5.44	1.48	0.2276
Akan	25.67	5.74		
Others	25.98	5.47		
Perceived relative Economic standing			7.86	0.0000
First Quintile	28.03	6.22		
Second Quintile	25.51	4.86		
Third Quintile	24.87	5.49		
Fourth Quintile	25.08	5.42		
Fifth Quintile	23.17	5.32		
**Employment**				
No	25.77	5.53	1.61	0.2047
Yes	25.19	5.50		

Table 4 features results from the multivariate regression model. In all, 3 models were used in assessing the relationship between psychological distress and both community and personal levels of trust, controlling for other explanatory factors. The first model expressed the relationship between only community level trust and psychological distress. The second model focused on the relationship between community level trust only and psychological distress. The third model expressed the relationship between the psychological distress and both community and personal trust while controlling for demographic factors. The results of the multivariate analysis showed that although community level trust was inversely related to psychological distress, the relationship was not significant (B = -0.1565, t = -1.59, p < 0.105). In model 1 (see Table 4), the results show that personal trust had a significant association with psychological distress. Personal trust was inversely related to psychological distress, meaning an increase in personal trust was associated with a significant decrease in psychological distress. Specifically, every unit increase in personal sense of trust, a 0.22 unit decrease in psychological distress was predicted (B = -.2151196, t = -2.88, p < 0.004).

**Table 4:** Multivariate model expressing the relationship between psychological distress and explanatory factors.

Variables	Model 1	P>[t]	Model 2	P>[t]	Model 3	P>[t]
Individual Trust	-0.21574	0.004			-0.202	0.010
Community Trust			-0.1565	0.105	0.002	0.982
**Locality**						
Usshertown						
Jamestown					-1.161	0.016
Agbogbloshie					-1.278	0.072
**Sex**						
Male						
Female					0.644	0.123
**Education**						
Primary						
Middle/JSS					-1.165	0.038
Secondary					-1.608	0.009
Higher					-3.006	0.003
**Marital Status**						
In Union					-0.305	0.486
Not in Union						
**Religiosity**						
Low						
Medium					0.612	0.340
High					0.291	0.639
**Ethnicity**						
Ga						
Akan					0.885	0.098
Others					1.010	0.118
Economic						
Perceived relative Economic standing					-0.841	0.000
**Employment**						
No						
Yes					-0.455	0.314

Variables included in the model: individual sense of trust, perceived economic standing, sex, age, marital status, religiosity, education, employment status and locality of residence. Model: R^2^ = 0.0804; Adjusted R^2^ = 0.0599; F (15, 673) = 545; p < .001

In model 3, other factors associated with psychological distress in this model were perceived economic standing, sex, education and locality of residence. A higher level of educational attainment was found to be negatively related to psychological distress. Higher educational attainment was significantly associated with lower levels of psychological distress. For example, respondents with the highest level of education attainment recorded the least psychological distress (B = -3.006885, t = -2.94, p < 0.003). Likewise, those with the least level of education recorded the highest psychological distress score (B = -1.165499, t = -2.07, p < 0.038). In terms of location, Usshertown recorded a higher level of psychological distress compared to Agbogbloshie and James Town. Perceived relative economic standing was significantly related with psychological distress. Respondents who rated themselves higher economically in the communities recorded significantly lower levels of psychological distress. Perceived relative economic standing was closely related to a higher sense of self efficacy.

## Discussion

This study examined the influence of community and individual sense of trust on psychological distress in three urban poor communities in Accra. Community sense of trust did not predict psychological distress. However, individual trust significantly predicted psychological distress. In addition to the main independent variables, sex, education, locality and relative perceived economic also significantly predicted psychological distress.

### 4.1 Sense of trust on psychological distress

The findings from this study showed that only personal sense of trust predicted psychological distress among urban poor residents in Accra. That is, a higher sense of personal trust was significantly associated with lower psychological distress. This finding is contrary to suggestions that communal living is the fundamental pathway to ensuring psychological well-being in collectivistic communities [40,46,47]. This study demonstrates that even in collectivistic urban poor communities, positive interpersonal attributes such as the ability to trust people attenuates the effect of psychological distress. There is evidence to show that in collectivistic contexts, people still maintain their sense of individuality and draw on their inter-personal psychological resources to buffer the stress of the collective [40]. In collectivistic and individualistic societies alike, individuals express individual, relational and collective selves, with each aspect of self becoming salient depending on specific personal and social circumstances[48]. Consistent with these arguments, the findings from the current study suggests that while mental health in urban poor communities is shaped by multi-level dynamics, personal and interpersonal level factors play more important buffer roles for psychological wellbeing than community level factors.

### 4.2 Demographic and socio-economic factors on psychological distress

Four of the demographic factors, namely sex, level of education, perceived relative economic standing and locality, predicted psychological distress. Formal education is generally associated with better health outcomes. Some researchers consider education to be a ‘social vaccine’ [49–51]. The educational effect on psychological distress can operate through access to social and labour-market resources [52]. In Ghana, individuals with higher education have access to better paid formal jobs and healthcare compared to those with no education [53]. These privileges may buffer individuals against the adverse effects of psychological distress in urban poor communities[54].

Perceived relative economic status was negatively associated with mental health in this study. Our finding are similar to those in Japan [55,56], and US [57], Perceived relative economic standing to some extent relates to the self-efficacy of community members. Respondents with a higher perceived relative economic standing had lower scores on the psychological distress scale. This implies that both inter (individual sense of trust) and intra personal attributes (perceived relative economic standing) were better predictors of psychological distress than community related variables. The model shows that perceived economic standing was the most significant predictor of psychological distress, while community sense of trust was the least significant predictor of psychological distress amongst the three main independent variables.

### 4.3 Limitations of the study

The study has a number of limitations. First, we cannot infer causality because the data we analysed was cross sectional. Also, the findings of the study cannot be generalized to the entire Ghanaian population. These findings are, however, relevant for rapidly expanding populations in major urban centres across the country. Additionally, other factors that measure sense of trust were not included in this study. For instance, the study did not directly measure the mitigating role of social institutions such as religious institutions on psychological distress.

### 4.3 Conclusion

This study presents evidence that more trusting individuals are significantly less likely to be psychologically distressed within deprived urban communities in Accra. Positive intra and inter individual level variables such as personal level trust and perceived relative economic standing significantly attenuated the effect of psychological distress in communities with high level neighbourhood disorder in Accra. Belonging to social groups where people can develop positive interpersonal relationships with each other may provide an avenue to buffer the effect of psychological distress in urban African contexts with rapidly growing populations and neighbourhoods with high levels of neighbourhood disorder.

## Supporting information

S1 Data The data for this analysis is contained in a stata file. (DTA)
